# The Surprising Role of Amyloid Fibrils in HIV Infection

**DOI:** 10.3390/biology1010058

**Published:** 2012-05-29

**Authors:** Laura M. Castellano, James Shorter

**Affiliations:** 1 Pharmacology Graduate Group, Perelman School of Medicine at the University of Pennsylvania, Philadelphia, PA 19104, USA; Email: lcast@mail.med.upenn.edu; 2 Department of Biochemistry and Biophysics, Perelman School of Medicine at the University of Pennsylvania, 804 Stellar-Chance Laboratories, 422 Curie Boulevard, Philadelphia, PA 19104, USA

**Keywords:** SEVI, amyloid, HIV infectivity, PAP85-120, SEM1, SEM2, microbicide

## Abstract

Despite its discovery over 30 years ago, human immunodeficiency virus (HIV) continues to threaten public health worldwide. Semen is the principal vehicle for the transmission of this retrovirus and several endogenous peptides in semen, including fragments of prostatic acid phosphatase (PAP248-286 and PAP85-120) and semenogelins (SEM1 and SEM2), assemble into amyloid fibrils that promote HIV infection. For example, PAP248-286 fibrils, termed SEVI (Semen derived Enhancer of Viral Infection), potentiate HIV infection by up to 10^5^-fold. Fibrils enhance infectivity by facilitating virion attachment and fusion to target cells, whereas soluble peptides have no effect. Importantly, the stimulatory effect is greatest at low viral titers, which mimics mucosal transmission of HIV, where relatively few virions traverse the mucosal barrier. Devising a method to rapidly reverse fibril formation (rather than simply inhibit it) would provide an innovative and urgently needed preventative strategy for reducing HIV infection via the sexual route. Targeting a host-encoded protein conformer represents a departure from traditional microbicidal approaches that target the viral machinery, and could synergize with direct antiviral approaches. Here, we review the identification of these amyloidogenic peptides, their mechanism of action, and various strategies for inhibiting their HIV-enhancing effects.

## 1. Introduction

Human immunodeficiency virus (HIV) causes acquired immune deficiency syndrome (AIDS) and has killed more than 25 million people [1,2,3,4,5]. Three decades after its initial identification, HIV endures as a global epidemic and aggressive public health threat: 33 million adults and children are living with HIV worldwide [5]. This problem is most acute in the developing world: ~68% of all adults and 90% of all children with HIV are in sub-Saharan Africa, and ~76% of all AIDS deaths occur in this locale [5]. The vast majority of these infections are acquired by heterosexual transmission [6,7,8]. Several factors dictate the efficiency of HIV transmission via the sexual route including the viral load, type of sexual practice, and susceptibility of the host [7,8]. Despite the growth and prevalence of the AIDS pandemic, HIV is an unexpectedly weak pathogen with low infectivity [9,10]. In fact, it is estimated that less than 0.1% of viral particles are infectious *in vitro,* since infectivity is limited by low viral attachment rates to host cells [11,12,13]. *In vivo* HIV transmission is also inefficient, occurring as infrequently as 1 in every 200 to 2000 acts of sexual intercourse [7,14]. However, high viral titers that occur during acute infection can enhance this rate ~10-fold [8], and further enhancements accrue with sexual practices connected with bleeding and lesions of the mucosal barrier as well as in the presence of other sexually transmitted diseases [14]. Semen is the principal vehicle for the sexual transmission of HIV, and *in vitro* semen can enhance the infection of physiologically relevant cell types, including primary macrophages and CD4+ T cells [15,16,17,18,19]. Thus, it was hypothesized that natural cofactors in seminal fluid could play a key role in HIV transmission by enhancing the efficiency of viral infectivity [16].

**Figure 1:**
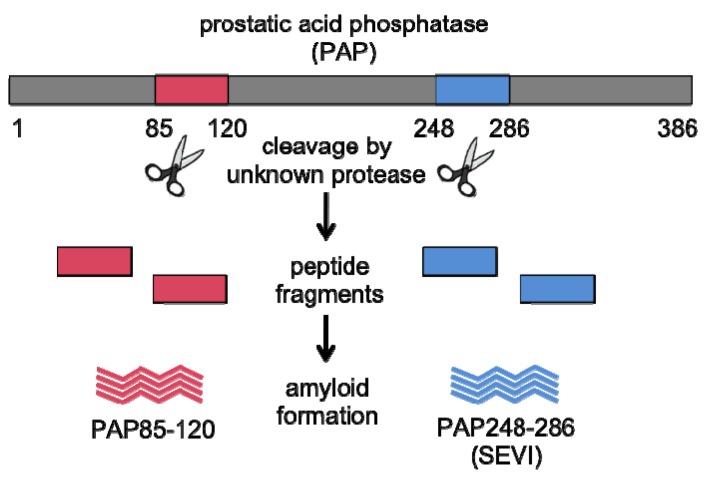
Proteolytic cleavage of prostatic acid phosphatase (PAP). Full-length PAP, a protein present at high concentrations in semen, undergoes proteolysis to form peptide fragments PAP85-120 and PAP248-286. These peptide products readily aggregate and assemble into amyloid fibrils in seminal fluid. Amyloid fibrils formed from PAP248-286 are termed SEVI (Semen-derived Enhancer of Viral Infection).

To isolate natural agents involved in the sexual transmission of HIV, Münch and colleagues created a library comprising all peptides and small proteins derived from human seminal fluid and screened this library for enhancers and inhibitors of HIV infection [16,18]. One fraction significantly enhanced HIV infection, and further analysis by mass spectrometry and sequencing revealed that the active fraction contained several peptides, each of which was a proteolytic fragment of prostatic acid phosphatase (PAP) [16], a protein highly abundant (1–2 mg/mL) in seminal fluid [20]. While these peptides differed in length, each mapped to the same region of PAP. The predominant peptide in the enhancing fraction corresponded to residues 248–286 of PAP and was isolated at a concentration of ~35 µg/mL (Figure 1) [16].

Unexpectedly, Münch *et al.* discovered that freshly dissolved solutions of chemically synthesized PAP248-286 were unable to augment HIV infection [16]. However, once the solutions became turbid after short-term storage or agitation, activity was restored, and in fact, the insoluble precipitate contained the active form [16]. Further biophysical analysis revealed that PAP248-286 fragments formed amyloid fibrils as indicated by increases in Thioflavin-T fluorescence, Congo red binding, and β-sheet content (Figure 1) [16]. These amyloid fibrils were termed SEVI (**S**emen-derived **E**nhancer of **V**iral **I**nfection) and were found to augment HIV infection by ~10^5^ fold under conditions of limiting virus, whereas soluble PAP248-286 had no effect [16]. The presence of SEVI reduced the number of virions required for productive infection from between 1000–100,000 to between 1–3 [16]. Indeed, the stimulatory effect of SEVI fibrils is greatest at low virus concentration, similar to the conditions observed in mucosal transmission of HIV, where relatively few virions traverse the mucosal barrier [16,19]. This remarkable effect is independent of viral coreceptor tropism [16], and the potency of individual human semen samples to boost infection correlates with levels of SEVI [15]. Moreover, SEVI also boosts the efficiency of retroviral transduction for viruses with diverse envelope proteins [21]. Intriguingly, previous studies have also implicated amyloids in the enhancement of viral infection. For example, Aβ40 and Aβ42 amyloid fibrils, which are involved in the pathogenesis of Alzheimer’s disease [22], stimulate HIV infection of microglia (by ~5–20-fold) and could contribute to HIV infection in the central nervous system of patients with HIV-associated dementia [23].

## 2. How Does SEVI Augment HIV Infection?

**Figure 2:**
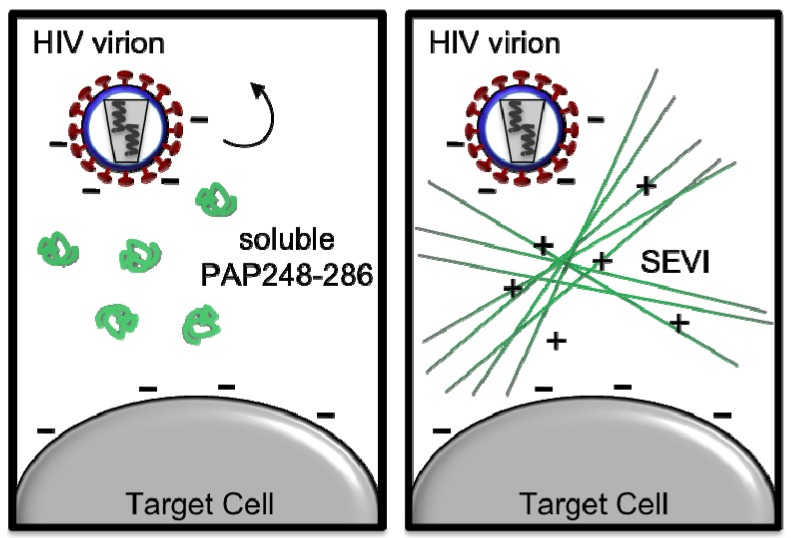
Semen derived Enhancer of Viral Infection (SEVI) amyloid fibrils augment HIV infectivity. In the non-amyloid form, soluble PAP248-286 has no effect on HIV infectivity, and electrostatic repulsion between the membranes of HIV virions and target cells limits the ability of virions to interact with target cells (left). Once assembled into amyloid fibrils, SEVI’s network of positive charges neutralizes this electrostatic repulsion, and SEVI fibrils capture HIV virions to promote cell attachment and fusion, thereby enhancing HIV infectivity (right).

The misfolding of proteins into a generic amyloid structure is a recurring facet of diverse neurodegenerative diseases [22,24,25,26]. Yet, in isolation many proteins can form amyloid fibrils, suggesting that amyloidogenesis is an integral part of polypeptide chemistry [22,27,28]. Indeed, amyloid fibrils have been exploited during evolution for various adaptive modalities including prion-based transmission of advantageous phenotypes, long-term memory formation, melanosome biogenesis, drug resistance and biofilm formation [29,30,31,32,33,34,35,36,37]. SEVI adopts a classic “cross-β” amyloid structure [16] in which β-sheets run orthogonal to the fibril axis [38,39,40]. The viral enhancing function of SEVI is dependent on its unique structural characteristics, as other generic “cross-β” amyloids enhance HIV infection with lower efficiency [16]. PAP248-286 contains a wealth of positively charged basic residues (8 of its 39 residues are either lysine or arginine; theoretical pI = 10.21). Thus, SEVI fibrils likely serve as a cationic bridge that simultaneously precipitates the virus onto the cell surface and decreases the electrostatic repulsion between the negatively charged surfaces of the virus and host cell (Figure 2) [19,41]. SEVI binds directly to both HIV virions and target cells [19], and this facilitates virion fusion much in the same way that synthetic cationic polymers promote retrovirus-mediated gene transfer [42,43]. Importantly, a mutant PAP248-286, in which all basic residues were replaced with alanine, was still capable of assembling into amyloid fibrils that could augment HIV infection, but activity was greatly reduced compared to SEVI [19]. NMR spectroscopy revealed that PAP248-286 is highly disordered when bound to membrane-mimicking micelles [44,45], whereas the majority of amyloidogenic peptides adopt an alpha-helical conformation upon micelle binding. It is predicted that some disordered regions present in monomeric PAP248-286 will be retained in mature SEVI amyloid fibrils [44,45,46]. Indeed, disordered segments that emanate from the surface of SEVI fibrils may increase fibril “capture radius” and could explain the enhanced ability of SEVI to promote HIV infection [44,45,47].

## 3. Current Strategies to Counteract SEVI-Mediated Enhancement of HIV Infection

The highly unanticipated role of SEVI in viral infection has opened a new window of opportunity. If we can inhibit the formation of SEVI amyloid fibrils, block their infection promoting properties, or even rapidly dissolve these fibrils, then we can drastically reduce HIV infection via the sexual route. Thus far, several agents have been identified that fall into each of these classes of anti-SEVI compounds. While this section focuses on agents that counteract SEVI’s effects, it should be noted that additional amyloid fibrils, which also augment HIV infection, have recently been identified [48,49]. Therefore, a truly effective agent should antagonize the stimulatory effects of all semen-derived amyloid fibrils.

Ultimately, agents that effectively block the ability of semen-derived amyloid to promote HIV infection might be developed into a microbicide, thus offering a preventative means to combat HIV transmission. A microbicide is an agent that reduces the infectivity of a pathogen. In this case, an anti-amyloid topical formulation might be developed for application either vaginally or rectally to prevent the very earliest stage of sexual HIV transmission [50,51]. Alternatively, methods to reduce initial amyloid formation in the seminal fluid of the male might also be explored. Ideally, the microbicide would be effective, safe, user-friendly and affordable to promote use in the developing world. The development of topical microbicides has proven challenging, and the vast majority of microbicidal compounds explored to date target the virus itself. However, mutations can rapidly arise that alter viral proteins and confer resistance to antiretroviral drugs [52]. Combinations of antiretroviral drugs tend to exhibit improved efficacy by functioning through multiple mechanisms of action [53]. By targeting host-encoded amyloid fibrils instead of the viral machinery, this issue of drug-resistant HIV variants could be circumvented, making amyloid fibrils a valuable and novel target for future microbicide development.

**Figure 3:**
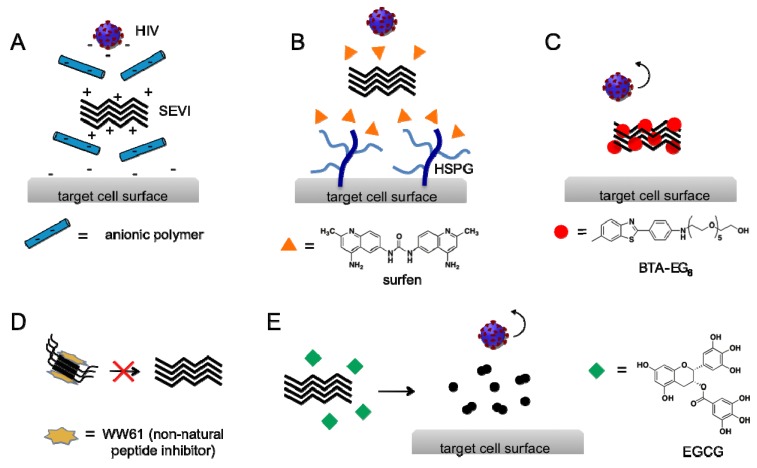
Mechanisms of action of anti-SEVI agents. (**A**) Anionic polymers shield the intrinsic positive charges of SEVI and inhibit the binding of SEVI to HIV virions; (**B**) Surfen, a heparin sulfate proteoglycan (HSPG) antagonist, inhibits interactions between SEVI and both HIV virions and target cells; (**C**) BTA-EG_6_ is a small molecule that engages SEVI’s cross-β structure by intercalating between β-strands; (**D**) WW61, a non-natural peptide inhibitor (Trp-His-Lys-chAla-Trp-hydroxyTic), inhibits SEVI fibrillization by binding to and obstructing growing fibril ends; (**E**) Epigallocatechin-3-gallate (EGCG) inhibits SEVI fibrillization and disaggregates pre-formed SEVI fibrils.

The most common class of anti-SEVI agents identified to date, shield the charged surface of the fibrils to block their infection boosting properties. Anionic polymers, which interfere with the binding of SEVI to HIV virions by shielding the intrinsic positive charges of SEVI, diminish SEVI-mediated enhancement of infection (Figure 3A) [19]. These polyanions, including heparin and various other glycosaminoglycans, have been investigated as HIV microbicides for decades, as they are also thought to exert direct anti-HIV activity by inhibiting the binding of the viral envelope glycoprotein, gp120, to its major cell surface receptor CD4 [54,55,56]. Unfortunately, such anionic polymers have proven unsuccessful in past clinical trials due to their poor bioavailability and induction of inflammatory responses in the genital tract, which instead augment HIV transmission by recruiting HIV-susceptible target cells to the genital mucosa [57,58]. Thus, more favorable options, which act through related mechanisms, are needed.

Aminoquinoline surfen, a small molecule heparan sulfate proteoglycan (HSPG) antagonist, exerts anti-SEVI effects through a slightly different mechanism (Figure 3B) [41]. It was initially hypothesized that SEVI bound to target cells through interactions with cell surface HSPGs [41]. Surfen antagonizes this interaction, and additional studies indicated that surfen also antagonized the interaction between SEVI and HIV virions [41]. Further, surfen possesses anti-inflammatory properties as an anaphylatoxin C5a receptor antagonist [59,60], making it a promising microbicide candidate.

BTA-EG_6_ (a hexa(ethylene glycol) derivative of benzothiazole aniline) is a small molecule capable of abrogating SEVI’s effects by binding to its “cross-β” structure (Figure 3C) [17]. BTA-EG_6_ is an amyloid-binding small molecule akin to the amyloid-diagnostic dye, Thioflavin-T, that intercalates between β-strands, and by doing so, inhibits the interactions of SEVI with both target cells and HIV virions and thus prevents SEVI-mediated enhancement of HIV infection [17]. Importantly, oligovalent derivatives of BTA exhibited an improved capability to attenuate SEVI-enhanced infection of HIV as compared to the corresponding monomer [61]. It is likely that these amyloid-binding agents form a bio-resistive coating on SEVI to hinder interactions with virions and target cells [17,61]. Indeed, similar amyloid-binding small molecules hold promise as potential therapeutics for various amyloid disorders. The ability of these small molecules to engage cross-β architecture and thereby prevent depletion of essential cellular factors by amyloid [62] has yielded promising outcomes in model systems. For example, Thioflavin-T suppresses pathological events caused by mutant metastable proteins and human β-amyloid in *C. elegans* [63].

**Figure 4:**
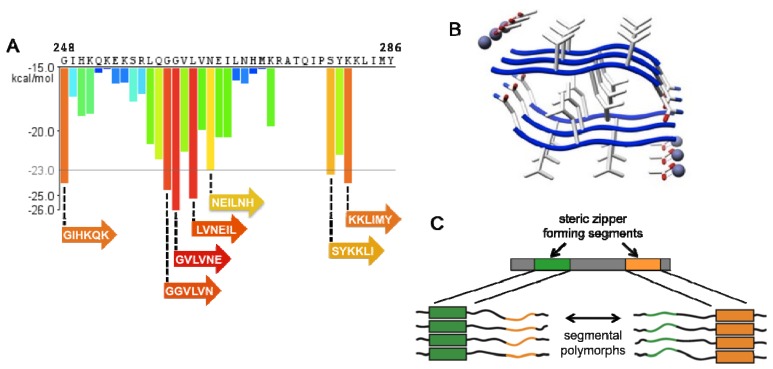
PAP248-286 has several regions of high amyloidogenic propensity. (**A**) Zipper DB [65] prediction of fibril-forming segments of PAP248-286. The propensity profile graph predicts hexapeptide segments (beginning at the indicated residue) that are highly likely to assemble into amyloid fibrils. Orange-red segments with energy values below the indicated threshold of −23 kcal/mol (gray line) are expected to form steric-zipper spines of fibrils. PAP248-286 contains seven hexapeptide segments of high amyloidogenic propensity; (**B**) The hexapeptide segment ^260^GGVLVN^265^ (energy = −24.5 kcal/mol) is one of the seven segments predicted to form fibrils. The steric-zipper structure of this hexapeptide has been solved (PDB ID: 3PPD). Acetic acid and zinc ions are shown associated with the structure and might indicate how SEVI engages phospholipids and tethers membranes [66]; (**C**) An amyloid-forming protein with two segments (green and orange) capable of forming steric zippers can exist as segmental polymorphs, in which each segment can form the amyloid core.

Another approach to target SEVI’s deleterious effects is through inhibition of amyloid fibril formation. Structure-based design of peptides comprised of non-natural amino acids has yielded a highly specific peptide inhibitor of PAP248-286 fibrillization [46]. This approach focused on the characteristic steric-zipper motif, common to amyloid-forming proteins, in which β-sheets pack together through interdigitation of their peptide side chains to form a highly self-complementary interface [39,64]. The G260 to N265 hexapeptide segment of PAP248-286, GGVLVN, is one of seven hexapeptides in PAP248-286 that is predicted by Zipper DB [65] to have high amyloid propensity (Figure 4A) [46]. The steric-zipper structure of GGVLVN was solved (Figure 4B) and used as a template to computationally design a peptide-based inhibitor, termed WW61, that would cap the growing ends of fibrils to inhibit amyloid assembly [46]. WW61 is a hexapeptide, Trp-His-Lys-chAla-Trp-hydroxyTic, which contains an Ala derivative, β-cyclohexyl-l-alanine (chAla) and a Tyr/Pro derivative, 7-hydroxy-(S)-1,2,3,4-tetrahydroisoquinoline-3-carboxylic acid (hydroxyTic), both of which are designed to increase contact area with the growing ends of SEVI fibrils. Remarkably, WW61 prevented full-length PAP248-286 from assembling into functional SEVI fibrils capable of promoting HIV infection (Figure 3D) [46]. While this breakthrough represents the first compelling example of a structure-based design of an amyloid inhibitor, there is no evidence that the WW61 peptide is active in blocking semen-mediated enhancement of HIV infection [46]. Indeed, WW61 is unlikely to inhibit the amyloidogenesis of PAP85-120, SEM1 and SEM2.

While this is a novel strategy to prevent SEVI assembly, SEVI fibrils are already formed and abundant in seminal fluid. Thus, inhibiting their initial formation would seem impractical in the male and futile in the female. Similarly, SEVI fibrils will persist in strategies that aim to coat the amyloid surface and prevent interactions with the virus or the cell surface or both. Thus, fibrils might still gain opportunities to promote viral infection. We suggest that a more desirable strategy would be to isolate agents that rapidly deconstruct SEVI fibrils and thereby eradicate structures that promote HIV infection. However, great difficulty lies in the unusual stability of the amyloid form, which resists proteases, protein denaturants, temperatures up to 98 °C, and 2% SDS [39,67,68]. Indeed, because of their exceptional stability, amyloid fibrils are difficult to degrade and are widely perceived to be intractable [69].

Despite their exceptional stability, a few, very select agents can reverse amyloid formation. EGCG (epigallocatechin-3-gallate), a potent antioxidant found in green tea, has shown widespread antifibrillogenic effects [70,71,72,73] and can even disassemble amyloid forms of islet amyloid polypeptide [74], α-synuclein [75], Aβ [75], and the yeast prion protein Sup35 [76]. By binding to exposed backbone sites in the unfolded regions of monomeric proteins, EGCG is proposed to divert aggregation pathways to instead favor the formation of off-pathway aggregates [71]. EGCG can inhibit formation of SEVI amyloid fibrils, and more importantly, it slowly remodels pre-formed SEVI fibrils over 1–2 days at non-toxic concentrations [77]. By targeting and remodeling these fibrils, EGCG strongly antagonizes the activity of SEVI (Figure 3E). In contrast to the initially proposed mechanism of action [71], EGCG was found to interact with the side chains of monomeric PAP248-286 (particularly lysine residues), leading to the formation of a covalently bound complex (Schiff base links with lysine residues) [78]. Notably, EGCG is extremely stable in acidic solutions, a condition resembling the vaginal environment [79]. These data, taken together, suggest that EGCG may be a favorable option for use as a topical microbicide component. Further studies were conducted to test the effects of EGCG on a cohort of 47 individual semen samples. Overall, EGCG reduced HIV infection by a median of 70.6% at a concentration of 0.4 mM [80]. Intriguingly, there was substantial heterogeneity in this result, as EGCG failed to have an inhibitory effect on 6 of the 47 semen samples. Thus, while EGCG holds promise as a potential microbicide supplement, these heterogeneities must be addressed.

In addition to its antifibrillogenic effects, EGCG also has direct antiviral activity in the absence of both SEVI and semen. By binding to cell-surface CD4 molecules on target cells, EGCG prevents attachment of the HIV glycoprotein gp120 to CD4 [81,82,83]. Interestingly, EGCG also exerts antiviral effects on both herpes simplex virus (HSV) [84] and Hepatitis C virus (HCV) [85]. In each of these cases, EGCG is believed to interact with either HSV or HCV glycoproteins, thus inhibiting cell entry by the virus. This direct antiviral activity provides further support for the use of EGCG as a component of future anti-HIV microbicides.

## 4. Amyloid Strains

A phenomenon that further adds to the complexity of searching for anti-SEVI and anti-amyloid agents is that amyloid fibrils can exist as polymorphs and form diverse conformational strains. Conformational variation among amyloid fibrils of a given protein can give rise to fibrils with distinct properties [86,87,88]. The biochemical basis for amyloid polymorphism likely stems, at least in part, from the diversity of steric-zipper amyloid spines that a single protein can adopt [89]. For example, PAP248-286 is predicted to contain seven different hexapeptide segments that are capable of forming steric zippers (Figure 4A). Thus, each of these segments could drive the assembly of amyloid structures that are distinct at the atomic level, resulting in a segmental polymorphism (Figure 4C) [89].

It is unclear whether each of the predicted hexapeptides present within PAP248-286 does indeed form a steric zipper. However, it is probable that SEVI fibrils can exist as multiple strains within seminal fluid. The presence of several SEVI strains could complicate the development of anti-SEVI inhibitors. For instance, WW61, the non-natural peptide inhibitor of SEVI assembly, was rationally designed to bind to the ^260^GGVLVN^265^ steric-zipper interface [46]. WW61 effectively inhibited PAP248-286 fibril formation, indicating that the ^260^GGVLVN^265^ plays a key role in driving SEVI formation under the specific assembly conditions employed [46]. However, this inhibitor might be ineffective against alternative segmental polymorphs (Figure 4A), which could assemble under different assembly conditions. Indeed, different assembly conditions can shift the relative populations of amyloid strains and generate distinct strain ensembles [76,88,89,90]. At present, the nature of the SEVI strain ensemble in seminal fluid is uncertain, and is likely to vary from individual to individual.

This concern also extends to EGCG, which is a strain-selective inhibitor of Sup35 fibrillization [76]. Such strain-selectivity could impact the ability of EGCG to exert antifibrillogenic effects against SEVI. In a study examining the efficacy of EGCG at reducing SEVI-mediated enhancement of HIV infection in a cohort of 47 individual semen samples, EGCG was only efficacious in 41 out of 47 samples, and substantial heterogeneity was observed within the results [80]. This heterogeneity might indicate that EGCG is a strain-selective antagonist of SEVI as well. Perhaps the most effective way to combat strain phenomena is to utilize combinations of inhibitors designed to target the entire spectrum of polymorphic strains [76,90,91,92,93].

## 5. PAP85-120 and SEM Amyloid Fibrils

**Figure 5:**
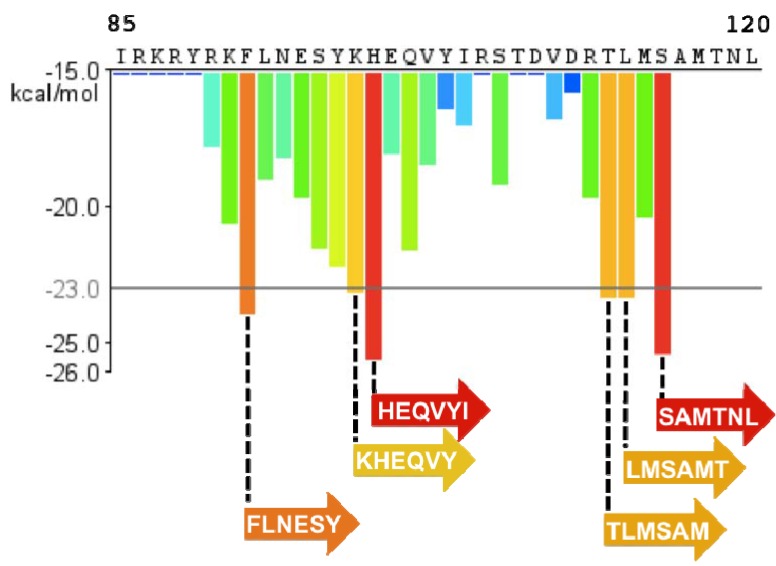
PAP85-120 has six regions of high amyloidogenic propensity. The Zipper DB histogram of PAP85-120 predicts six hexapeptide segments that are highly likely to form steric-zipper spines.

After identifying SEVI, Münch and colleagues continued analysis of their semen-derived peptide library [48]. In particular, they noted an additional fraction that promoted HIV infection. Remarkably, this fraction was identified to be an N-proximal fragment of PAP that comprised residues 85-120 (PAP85-120), and this peptide was isolated at a concentration of ~39 µg/mL (Figure 1) [48]. The discovery of a second peptide fragment that corresponded to the same precursor protein was unanticipated, especially since the library contained several thousand species. Like SEVI, PAP85-120 exists as amyloid fibrils in seminal fluid, and these fibrils promote HIV infection but are slightly less efficient [48]. Also like PAP248-286, PAP85-120 is highly cationic (8 of its 36 residues are either lysine or arginine; theoretical pI = 9.99) implying that PAP85-120 and SEVI act through a similar mechanism to boost HIV transmission. Six hexapeptides within PAP85-120 are predicted by Zipper DB [65] to be amyloidogenic, which suggests that PAP85-120 might also form different amyloid strains (Figure 5). Notably, SEVI and PAP85-120 fibrils cooperate to exert additive HIV enhancing effects in cell culture [48].

**Figure 6:**
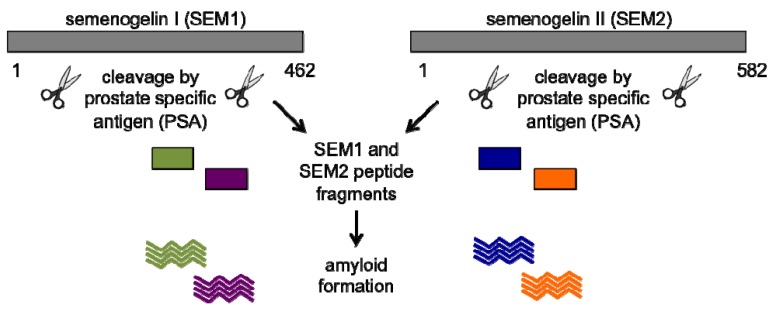
Proteolytic cleavage of semenogelin 1 (SEM1) and semenogelin 2 (SEM2). Full-length SEM1 and SEM2, the major components of the semen coagulum, are proteolytically cleaved by prostate specific antigen (PSA) to yield several peptide fragments. These peptide products readily aggregate and assemble into amyloid fibrils in seminal fluid.

**Figure 7:**
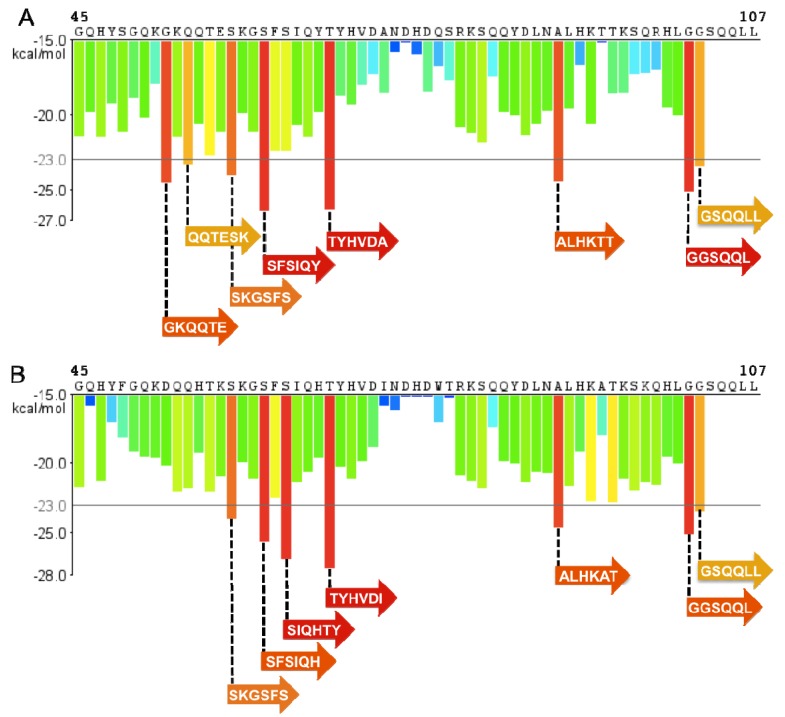
SEM1 and SEM2 have several regions of high fibrillization propensity. (**A**) The Zipper DB histogram of SEM1(45-107) indicates eight hexapeptide segments with energies below -23 kcal/mol that are predicted to form fibrils; (**B**) The Zipper DB histogram of SEM2(45-107) indicates seven hexapeptides segments that fall below the energy threshold and are thus predicted to form fibrils.

The discovery of two positively charged fibrillar structures that augment virion fusion and infection led to the hypothesis that other positively charged factors might exist in semen that could also enhance HIV transmission. To test this hypothesis, pooled seminal fluid was depleted of positively charged factors and this resulted in a complete loss of its ability to augment HIV infection [49]. When the identity of these depleted cationic factors was subsequently determined, SEVI was surprisingly not detected. Rather, proteolytic fragments from two homologous semenogelin proteins, SEM1 and SEM2, were identified (Figure 6) [49]. Semenogelins (SEMs) are proteins originating from the seminal vesicles that together with fibronectin comprise the major components of the semen coagulum [94,95]. SEM proteins are rapidly cleaved after ejaculation by the chymotrypsin-like serine protease, prostate specific antigen (PSA), at highly specific sites [96,97]. This cleavage event is associated with liquefaction of the semen coagulum, which is important for the release of motile spermatozoa and thus male fertility [94,95]. The SEM1 and SEM2 peptides detected from this experiment corresponded to PSA-generated proteolytic fragments, and remarkably, these peptide fragments all formed amyloid fibrils capable of promoting HIV infection to varying extents [49]. Like SEVI and PAP85-120, the SEM peptides are rich in lysine and arginine residues and carry a net positive charge at neutral pH (pIs of SEM peptides studied range from 8.16 to 10.12). The SEM1(45-107) and SEM2(45-107) peptides each contain multiple predicted regions of high fibrillization propensity (Figure 7), implying that different amyloid strains may exist for these peptides as well.

Interestingly, semen samples from patients with ejaculatory duct obstruction (EDO) were also analyzed. EDO patients are naturally deficient in SEMs, since duct obstruction prevents contents of the seminal vesicles from reaching the ejaculate. As expected, these semen samples lacked viral infection-enhancing activity [49]. Unexpectedly, however, levels of full-length PAP were elevated in EDO samples, while levels of the SEVI precursor peptide PAP248-286 were diminished [49]. Because semen from EDO patients lacks secretions from the seminal vesicles, these data suggest that the proteolytic activity responsible for generating the SEVI peptide from full-length PAP is diminished in semen from EDO patients. The identity of the protease that releases PAP248-286 from its full-length precursor is undetermined, but these data indicate that the protease may reside in the seminal vesicles. The identification of this protease will be valuable, since a protease inhibitor could be a useful tool to prevent the release of PAP248-286 and PAP85-120 peptides from their full-length precursor. Such a strategy could also be employed to inhibit PSA, thus blocking the cleavage of amyloidogenic SEM peptides from full-length SEM1 and SEM2.

The identification of numerous amyloidogenic peptides in seminal fluid is somewhat surprising. The presence of multiple peptides assembled into amyloid fibrils in semen suggests that they are potentially functional rather than disease-associated. Indeed, in humans, amyloid conformers have been captured for various beneficial purposes, including melanosome biogenesis and the natural storage of peptide hormones in pituitary secretory granules [29,30,32,98,99]. Does the make-up of seminal fluid favor the formation of amyloid fibrils? Studies of SEVI, PAP85-120, and SEM amyloid fibrils have indicated that high peptide concentrations and extensive, non-physiological levels of agitation are required to induce amyloid formation *in vitro*. However, when the SEVI precursor peptide PAP248-286 was assembled into amyloid fibrils in the presence of seminal plasma, fibrillization was significantly enhanced and accelerated [100]. Seminal plasma drastically reduced the lag time for SEVI fibrillization and eradicated the requirement for agitation. This finding indicates that the diversity of molecules in seminal plasma, especially anionic buffer components, have a drastic impact on fibrillization and can potentially explain the strong presence of amyloid fibrils in seminal fluid. Furthermore, semen contains high concentrations of Zn^2+^ cations (low mM concentrations), which can inactivate seminal proteases and protect amyloid fibrils from proteolytic degradation [100]. In fact, a positive correlation exists between HIV-infection enhancement and zinc concentration in semen, indicating that Zn^2+^ cations likely stabilize semen-derived amyloid fibrils, thus increasing their capacity to augment infection [101].

## 6. A Physiological Role for Semen-Derived Amyloid Fibrils?

While the role of these naturally occurring semen-derived amyloidogenic peptides in HIV infection has been well defined, one crucial question is still unanswered: what is the adaptive role of these amyloid species? It has been postulated that these amyloid fibrils may play a physiological role in promoting fertilization, especially since numerous commonalities exist between virion-target cell fusion and sperm-egg fusion [102]. Thus, semen-derived amyloid fibrils may facilitate the attachment and fusion of sperm with an oocyte much in the same way that SEVI and other semen-derived amyloid fibrils promote HIV virion binding and fusion with target cells. The most obvious commonality between the two processes is that they occur in the same anatomical context, and semen is utilized as a vehicle to transport both sperm and HIV. Another striking parallel between the two processes is that the HIV viral envelope and sperm share the same basic plasma membrane structure consisting of a phospholipid bilayer embedded with vital glycoproteins and lipids [102]. Furthermore, both processes proceed by an identical series of steps involving cell binding, fusion, and penetration. First, sperm and HIV must attach and bind to their target cells, and both carbohydrates and electrochemical interactions are crucially involved in this step [102]. Just as polyanions have been shown to block SEVI-mediated enhancement of HIV infection by inhibiting HIV cell entry [19,103], several polyanions have also been shown to obstruct sperm-oocyte interactions [104,105], further illustrating the similarities between these processes. Following cell attachment, membrane fusion must occur in both fertilization and HIV infection. The fusion of sperm and oocyte membranes, as well as HIV and target cell membranes, is an energetically unfavorable process that requires cooperative protein-protein interactions [104,106,107]. Finally, overlapping signal transduction pathways are essential for both sperm capacitation (a process through which sperm acquire fertilization capacity) and the post-entry stages of HIV replication and maturation [102,108,109].

Because of the apparent parallels between mammalian fertilization and HIV infection, it is certainly plausible that SEVI and other amyloids may play a role in the fertilization process. Further evidence stems from the fact that spermatozoa can be stained with the amyloid-binding dye Congo Red, indicating that amyloid fibrils may be bound to these motile sperm cells [110]. Additionally, immunostaining for SEM1 and SEM2 has revealed that SEM fragments bind to the posterior head, midpiece, and tail of ejaculated spermatozoa [94,111,112]. However, it is unclear whether or not this result involves SEM amyloid fibrils or non-amyloid SEM fragments. If semen-derived amyloid fibrils are indeed bound to spermatozoa, this will further suggest a mechanism by which amyloid fibrils are involved in fertilization.

## 7. Looking to the Future

SEVI represents a novel microbicide target. Thus, additional means of counteracting SEVI-mediated enhancement of HIV infection are being explored, and similar approaches should also be investigated to counteract the effects of PAP85-120, SEM1, and SEM2 amyloid fibrils. One strategy is to use conformation-specific antibodies that specifically recognize aggregated forms of proteins. For amyloid fibrils to form, a small peptide segment within the aggregated protein must form a tight, self-complementary interface with an identical segment [64,65,113], and this natural process can be exploited to create amyloid-specific antibodies. Gammabodies, or grafted amyloid-motif antibodies, have been constructed by taking these amyloidogenic peptide segments and grafting them into complementarity determining regions (CDRs) of antibodies [114]. Amyloidogenic segments of the Aβ42 peptide, which is associated with Alzheimer’s Disease, have been used to generate gammabodies that recognize Aβ42 amyloid fibrils and soluble oligomers with nanomolar affinity [114]. These grafted motifs engage in homotypic interactions with the corresponding peptide segments within aggregated Aβ conformers, and this binding has been shown to neutralize the toxicity of Aβ oligomers and fibrils [114]. This approach could also be utilized to construct gammabodies that specifically recognize SEVI amyloid fibrils. By binding to SEVI with high affinity, gammabodies could inhibit the infection enhancing property of these amyloid fibrils and therefore serve as a highly selective and effective anti-SEVI agent.

A second future approach involves developing a novel transmission-blocking HIV vaccine to protect against HIV infection via the sexual route. SEVI binds HIV virions directly through electrostatic contacts [18], and virus-SEVI complexes could present a novel antigenic target for such a vaccine. Whether or not these virus-SEVI complexes can elicit virus-neutralizing antibodies is being explored [115]. The advantage of this approach is that an elicited immune response would recognize the authentic form of HIV, as it is found in semen, thereby inhibiting sexual transmission of the virus.

## 8. Concluding Remarks

Despite the wealth of evidence illustrating the role of semen-derived amyloid fibrils in HIV transmission, the actual presence and proviral activity of these fibrils has been questioned. One study reported that the infection enhancing activity of SEVI fibrils formed *in vitro* was neutralized in the presence of 1% seminal plasma [116]. This effect was attributed to the natural degradation of the PAP248-286 peptide by seminal proteases [116], thus calling into question whether such fibrils could actually form naturally in seminal fluid. However, amyloid fibrils are readily identified in fresh human seminal fluid using transmission electron microscopy [101]. A related study found that seminal plasma protects CD^+^ T cells from infection with both X4 and R5 tropic HIV due to semen mediated reduction in CD4 receptor expression [117], and seminal plasma also inhibits the capture and transmission of HIV to CD^+^ T cells mediated by DC-SIGN [118]. It is clear that both proviral and antiviral factors in semen modulate HIV infectivity, and it will be important to determine how the interplay between these opposing factors can be manipulated to prevent HIV infection.

The relative contributions of SEVI, PAP85-120, and SEM amyloid fibrils, as well as amyloid strain phenomena, in semen-mediated enhancement of HIV infection have yet to be determined. However, defining these contributions will be important in the design and search for agents that can abrogate the deleterious effects of these amyloid fibrils. Perhaps the most useful agents to be discovered are those that target amyloid fibrils in general, rather than specific inhibitors of SEVI, PAP85-120, or SEM amyloid fibrils. Ultimately, agents that can efficiently disaggregate or degrade these amyloid fibrils could be developed into a vaginal microbicide, thus reducing amyloid-mediated enhancement of HIV infectivity and offering a preventative strategy to combat HIV infection via sexual transmission. In this context, it will be interesting to explore amyloid disaggregases that can rapidly dissociate amyloid forms within a few minutes [69,119,120,121]. Here, it will be important to isolate conditions where disaggregases completely eliminate amyloid forms rather than yielding fragmented fibrils, which might enhance HIV infection [122].

Until recently, HIV prevention methods have lacked credibility. Data from various trials showed limited decreases in HIV incidence [123]. Moreover, the explanation for some publicized successes is not entirely clear [124,125,126]. However, prevention based on antiretroviral drugs incorporated into microbicides has recently been more successful [127,128,129]. For example, application of a microbicide gel containing 1% tenofovir (a nucleotide analogue reverse transcriptase inhibitor) into the vaginal vault within 12 hours of intercourse reduced overall acquisition of HIV by 39–54% [127,129]. The idea of targeting host protein conformers (semen-derived amyloid fibrils) is fundamentally different from traditional microbicidal approaches that target HIV itself. This strategy might be less susceptible to HIV escape variants, which can rapidly evolve and evade therapies that directly target components of the viral machinery [130,131,132]. We anticipate that anti-amyloid strategies will synergize with direct antiviral strategies, such that microbicides containing anti-viral agents and anti-amyloid agents (especially amyloid disaggregases) could display enhanced efficacy. Combination therapies have been extremely successful in treating HIV [133,134], and combinations should also be considered for HIV prevention [123,135]. Indeed, combination strategies might be potentiated by simultaneously targeting components of the host and viral machinery. Thus, addition of an effective anti-amyloid system could even enhance the efficacy of the already promising tenofovir microbicide gel [127]. Finally, the numerous commonalities between fertilization and viral infection (involving sperm and HIV membrane structure, cell binding, fusion, and signaling pathways) could provide a framework for developing dual-action contraceptive microbicides that possess both antimicrobial and contraceptive properties.
